# Misidentification by farmers of the crop varieties they grow: Lessons from DNA fingerprinting of wheat in Ethiopia

**DOI:** 10.1371/journal.pone.0235484

**Published:** 2020-07-07

**Authors:** Moti Jaleta, Kindie Tesfaye, Andrzej Kilian, Chilot Yirga, Endeshaw Habte, Habekiristos Beyene, Bekele Abeyo, Ayele Badebo, Olaf Erenstein

**Affiliations:** 1 International Maize and Wheat Improvement Center (CIMMYT), Addis Ababa, Ethiopia; 2 Diversity Arrays Technology (DArT), Bruce, Australia; 3 Ethiopian Institute of Agricultural Research (EIAR), Addis Ababa, Ethiopia; 4 Central Statistical Agency (CSA), Addis Ababa, Ethiopia; 5 International Maize and Wheat Improvement Center (CIMMYT), Texcoco, Mexico; International Institute of Tropical Agriculture (IITA), MALAWI

## Abstract

Accurate identification of crop varieties grown by farmers is crucial, among others, for crop management, food security and varietal development and dissemination purposes. One may expect varietal identification to be more challenging in the context of developing countries where literacy and education are limited and informal seed systems and seed recycling are common. This paper evaluates the extent to which smallholder farmers misidentify their wheat varieties in Ethiopia and explores the associated factors and their implications. The study uses data from a nationally representative wheat growing sample household survey and DNA fingerprinting of seed samples from 3,884 wheat plots in major wheat growing zones of Ethiopia. 28–34% of the farmers correctly identified their wheat varieties. Correct identification was positively associated with farmer education and seed purchases from trusted sources (cooperatives or known farmers) and negatively associated with seed recycling. Farmers’ varietal identification thereby is problematic and leads to erroneous results in adoption and impact assessments. DNA fingerprinting can enhance varietal identification but remains mute in the identification of contextual and explanatory factors. Thus, combining household survey and DNA fingerprinting approaches is needed for reliable varietal adoption and impact assessments, and generate useful knowledge to inform policy recommendations related to varietal replacement and seed systems development.

## Introduction

Crop varietal identification in farmers’ fields has long relied on elicitation of farmers’ own identification of the varieties in household surveys. Ethiopia is no exception, and several adoption and impact assessments of improved crop varieties have been conducted there relying on farmers’ elicitation [1–4]. Farmers might report their variety with a specific name, possibly corresponding with names listed in the national variety registry or a locally adapted name. In some cases, farmers might not report a specific name but use generic terms as ‘*improved*’ or ‘*local*’ or refer to the presumed origin. These farmer-reported names are typically taken at face value and subsequently used to estimate adoption rates and associated impacts. Of late, the reliability of such varietal information collected based on farmers’ elicitation has been increasingly questioned by the advent of DNA fingerprinting [5–12].

Farmers misidentification of their crop varieties is an example of measurement error. Survey measurement error through such farmer recall can be thought to represent simple misreporting correctable through improved measurement [13], like now possible with DNA fingerprinting. Correcting such varietal identification error is important in relation to varietal development and dissemination, including replacing old varieties with better performing new ones [14] and documenting the adoption of improved crop varieties.

Varietal misidentification might also reflect misperceptions that materially affect the respondents’ decisions under study [13]. Crop varieties variously respond to crop management (e.g. fertilization, timeliness) with important productivity and profitability considerations. Misidentification may thus result in production inefficiencies. For instance, farmers may apply fertilizer to what they think are improved varieties but are in fact unimproved; or alternatively, may fail to apply fertilizer and other agronomic practices to what they think are local varieties but are in fact improved. Farmers' technology adoption decisions can indeed exhibit strong input complementarity for some inputs [15]. Food security considerations at household and national levels are of particular concern in relation to tracking and replacing specific varieties susceptible to diseases and insect pests. Smallholder farmers in Ethiopia rely on wheat for subsistence and livelihood security. Farmers can replace stress susceptible varieties with more tolerant or resistant ones, like in the case of wheat rust epidemics [16]. Knowing the prevalence of susceptible wheat varieties also is key to assist policy makers target limited stocks of fungicide during wheat rust alerts in countries such as Ethiopia [17] and thereby the effectiveness of policy intervention [15].

Misidentification may also have direct economic impacts on how markets function. For example, grain marketing and processing may be variety-dependent as some varieties possess special traits preferred by consumers or processing industries (e.g. bread vs durum wheat varieties in Ethiopia). Misidentification may originate along the seed supply, including mixing with other varieties at seed production, processing and marketing [18], and information bottlenecks in seed delivery and extension [19].

One may expect varietal identification to be more challenging in the context of developing countries with limited literacy and education, with informal seed systems and saving and recycling of seed [2, 20–22]. In Ethiopia wheat seed is widely recycled and varieties are not proprietary, thereby reducing incentives for seed sellers to engage in possible fraud. Moreover, seeds from informal markets/sources could be heterogeneous and with diverse local names. Official variety names may also be complicated for illiterate farmers (e.g. scientific names, numbers or names with locally unknown meanings), and farmers may rebrand varieties with easier local names, e.g. describing varietal features or one that is more easily remembered [23].

The contribution of this paper is two-fold. First, we establish the extent of crop varietal misidentification based on nationally representative data from a developing country. We do so using the case of wheat in Ethiopia and contrasting estimates of varietal identification using conventional household surveys based on farmers’ elicitation with results from DNA fingerprinting (DNA FP) methods. Second, we identify some of the factors influencing farmers’ ability to correctly identify the crop varieties they grow. The findings have various implications, particularly for breeding programs, varietal replacement and crop protection strategies in developing countries in general, and wheat in Ethiopia in particular. Importantly, a better understanding of these two facets provides a foundation for subsequent explorations of the economic implications of seed misidentification.

## Data and methods

### Reference library

In varietal identification using DNA FP, building a comprehensive reference library is key. From 1960s to 2016, a total of 133 improved wheat varieties were released in Ethiopia. From the 133 released varieties, breeder seeds of 111 varieties were collected from the original research centres that developed and released these varieties. Breeder seeds for the remaining 22 varieties were not available for collection due to their old age and inability to trace viable sources. In the survey, only 1.2% of the farmers reported that they grew eight of these 22 varieties, which is a small proportion to affect the overall analysis results. For the collected breeder seeds, DNA was extracted from each variety at Holeta National Agricultural Biotechnology Research Center (NABRC), Ethiopia. With careful and proper tags, the DNA extracted from these breeder seeds were sent to the Diversity Arrays Technology (DArT) in Australia to build a reference library to be used for any sequencing and identification purposes of wheat samples collected from Ethiopia.

### Sampling, data collection and generation procedure

Data used in this study for variety identification were collected from two sources: (1) DNA FP of grain samples collected from randomly selected wheat plots, and (2) survey data from households operating the wheat plots from where the grain samples were collected for DNA FP. Ethiopia’s Central Statistical Agency (CSA) led the data collection with technical support from the Ethiopian Institute of Agricultural Research (EIAR). Though there is no permanent internal review board at CSA, before the survey was conducted, the survey instruments were assessed for their compatibility on ethical standards by a team of experts from the Agriculture, Natural Resources and Environmental Statistics Directorate (ANRESD) at CSA. The survey data were collected by experienced and well-trained enumerators speaking the local languages fluently and hired by CSA. Before each interview started with a sample household head (or respondent), the enumerators explained the purpose of the study and the anonymity of all information they provide. Then enumerators asked respondent’s consent to continue with the interview. All the sample households responded during the survey passed through this procedure and provided their full consent orally.

CSA has a long experience in collecting crop production data through its Agricultural Sample Survey (AgSS). For the same purpose, CSA uses Enumeration Areas (EA) as a minimum sampling unit. By considering the four major regional states (Oromia, Amhara, SNNPR, and Tigray) together producing over 95% of Ethiopia’s wheat, and the major administrative zones producing wheat in these four regional states, CSA randomly selected 420 EAs for wheat data collection (Table 1).

**Table 1:** Sample distribution by region and zone, Ethiopia 2016/17 (total n = 3,884).

Tigray		Amhara		Oromia		SNNPR^†^	
Zone	n	Zone	N	Zone	n	Zone	n
Central Tigray	122	Awi	53	Arsi	201	Alaba	59
Eastern Tigray	170	East Gojjam	243	Bale	107	Amaro Special	6
South Tigray	195	North Gondar	53	East Hararghe	73	Bench Maji	6
Western Tigray	12	North Shewa	116	East Shewa	112	Burji Special	8
		North Wollo	168	East Wollega	49	Dawuro	25
		Oromiya- Zone	5	Guji	7	Derashe Special	3
		South Gondar	129	Horro Guduru Wollega	122	Gamo Gofa	69
		South Wollo	179	Ilu Abba Bora	35	Gedeo	1
		Wag Himra	91	Jimma	78	Gurage	88
		West Gojjam	82	North Shewa	81	Hadiya	193
				Qellem Wollega	38	Kefa	31
				South West Shewa	148	Kembata Tambaro	148
				West Arsi	70	Konta	10
				West Hararghe	13	Sheka	1
				West Shewa	88	Silit	184
				West Wollega	16	South Omo	30
						Wolaita	15
						Yem Special	152
Zonal total	499 (13%)		1,119 (29%)		1,238 (32%)		1,028 (26%)

^†^ SNNPR stands for the Southern Nations, Nationalities and Peoples Regional State in Ethiopia.

In each randomly selected EA, again a maximum of 10 wheat plots were randomly selected from the available wheat plots during the 2016/17 main cropping season. It is worth noting that the number of wheat samples in less wheat potential EAs could be lower due to lack of enough number of wheat plots within the specific EA. From these randomly selected wheat plots, wheat grain samples were collected from a randomly identified *4m-by-4m* quadrant within the sample plots. Plot owners were also interviewed to get household and farm level data including names of the wheat varieties grown on the sample plots. Both the survey data and collected grain samples were transferred to Holeta NABRC for data entry and DNA extraction, respectively. To accurately trace the survey and the associated DNA FP data, both the survey questionnaire and the grain bags for sample collection were tagged with the same unique barcode for each wheat plot. Extra copies of the unique barcodes were kept in the grain bags for use during DNA extraction and shipping the DNA samples to DArT for DNA fingerprinting.

The crop-cut data for yield estimation was taken from a *4m-by-4m* sub-plot randomly selected within a specific wheat plot. The wheat plots were also randomly selected from the existing list of wheat plots in a pre-selected EA. Grain obtained from the 16 m^2^ sub-plot area was dried to at least 12.5% moisture content before the final weight measurement was taken. From the harvested and dried grain, after measurement, 250 gm was taken for DNA extraction to the Holeta NABRC. Part of the grain from each sample was grinded and DNA was extracted following a standard Zymo kit protocol at NABRC. The extracted DNA samples were shipped to the Diversity Arrays Technology (DArT) in Australia for DNA fingerprinting/sequencing following DArTseq method. For genotyping by sequencing DArTseq, a combination of a DArT complexity reduction methods and next generation sequencing platforms was used [24, 25]. The remaining grain in each bag was kept in a cold room as backup and for future use. Then, farmers were also interviewed using a survey instrument to elicit the name of the specific variety and other agronomic practices used on each of their plots from which crop-cuts were taken. The survey data and DNA sequenced results obtained from DArT were merged for analysis using the unique identification barcodes on both entities.

### Survey and DNA sequenced data

During the 2016/17 data collection, 3,884 sample wheat plots were surveyed and grain samples were collected for DNA extraction and fingerprinting. A total of 3,771 wheat samples were genotyped against the total 111 unique varieties in the reference library, with 123 (3%) samples dropped for various reasons, including DNA quality. Through DNA FP, 3,543 (94%) of the samples were identified with specific wheat variety names against the existing reference library using a minimum 70% purity level set by DArT as a cut-off point in DNA sequencing. Using data from sequencing of DNA of grain samples obtained from farmers’ fields, 45 unique wheat varieties were identified. From the 3,543 samples identified using DNA FP, variety names from DNA FP and farmer reporting matched for only 989 (i.e., 27.9%) of the observations (Table 2). 228 samples (6%) remained unclassified by DNA FP–i.e. these samples do not map against any identified variety in the reference library. This could be the case for local varieties or (old) improved varieties not included in the library, whereby farmer identification could be potentially correct. It could also include cases of impure varieties and/or potentially still farmer misidentified. Therefore, we added the 6% to the correctly matched set to provide the potential upper range of correctly identified varieties, i.e., 28–34%.

**Table 2:** Descriptive statistics for farmer reported wheat variety names and DNA sequenced data, Ethiopia 2016/17.

Category	Frequency	*%*
*Farmer reported names (n)* ^a^	3,884	*100*.*0*
Farmer reported variety with specific name ^b^	2,222	*57*.*2*
• Name in the variety registry ^c^	1,957	*50*.*4*
• Name not in the variety registry ^d^	265	*6*.*8*
Farmer reported with generic name (i.e. non-specific name) ^e^	1,662	*42*.*8*
• Reported as ‘*local variety but I don’t know the name*’ ^f^	1,092	*28*.*1*
• Reported as ‘*improved variety but I don’t know the name*’ ^g^	570	*14*.*7*
*DNA sequenced samples (n)*	3,771	*100*.*0*
Uniquely DNA identified (at >70% purity level against reference library)	3,543	*94*.*0*
Not identified by DNA FP	228	*6*.*0*
Match between DNA identified & farmer reported name	989	*27*.9*

a = b+e; b = c+d; e = f+g.

*27.9% based on DNA identified samples only (i.e. 989/3543).

### Empirical approaches

A binary Logit model was used in assessing factors explaining the mismatch between variety names reported using farmers’ elicitation and identified by DNA sequencing. Referring to varietal names identified by the DNA sequencing, a value of 1 is assigned to the cases where farmers accurately identified varieties they grew and 0 for those not. Household characteristics, seed sources from where the initial seeds of the varieties were obtained, and plot characteristics are considered in the regression analysis. To further explore the mismatch between varietal names, we sub-categorized the mismatched samples into four: (1) those which didn’t match but reported by farmers with a specific name in the national variety registry, (2) those reported by farmers with specific name but names are not in the registry, (3) farmers reported the variety using generic name as *‘Improved variety but specific name not known’*, and (4) *‘Local variety but specific name not known’*. Taking samples with the exact match as a reference, we applied a multinomial logit model to assess variables explaining the likelihood that varietal identification using farmers’ elicitation falls in any one of these mismatched categories. With *J* possible categories where a farmer-reported varietal name could fall under, a multinomial logit model is specified as:

$$P\left(y=j|X\right)=\frac{\exp\left(X\beta_{j}\right)}{1+\sum_{m=1}^{J}\exp\left(X\beta_{m}\right)}\;;\;where\;j=0,1,2,\ldots ,J\;and\;m\neq j$$ (1)

Where *P*(*y* = *j|X*) is the probability that farmers’ elicitation happened to be matching with DNA FP result or taking one of the four mismatch cases stated above. *X* is a vector of covariates affecting farmers’ varietal knowledge, and *β* is a vector of parameters to be estimated.

When reporting wheat grown, in 50% of the cases, farmers’ elicitation picked specific varietal names in the national registry (Table 2). However, 309 farmers reported a variety with a name of a recently released ones where the variety, as identified by DNA FP, was actually older than what the farmers reported. With the assumption that recently released varieties have better traits to resist rust and show better yield performance, the report is ‘*false’* but ‘*positive’* as it picked names of recently released varieties when the actual variety was an old release. On the other hand, 436 farmers reported a variety with a name of an old variety when the actual variety grown was a recent release, that is a ‘*false’* report and ‘*negative’* in picking an old variety name when the variety was actually a recent release. Such a mix-up of variety names in farmers’ elicitation, and mixing names of varieties with different varietal age has a consequence on varietal replacement strategies farmers follow. To explore this, we used a multinomial Logit estimation considering accurately identified varieties as a reference.

Accurate varietal identification is also important to estimate the average age of varietal replacement. To get this estimate for wheat varieties grown in Ethiopia, we used the following equation stated in [26].

$$WA_{t}=\sum_{i=1}^{I}p_{it}R_{it}$$ (2)

Where *WA_t_* is area weighted average age of a variety at a given *t, p_it_* is the proportion of area sown to variety *i* in year *t*, and *R_it_* is the number of years (at time t) since the release of variety *i*.

## Results

### Variety-level correspondence between farmer reporting and DNA fingerprinting

DNA FP identified 45 improved wheat varieties, 38 of which are bread and 7 durum wheat (Table 3). In terms of frequency, *Kakaba*, *Kubsa*, *Danda’a*, *Digalu* and *Bobicho* are the top five varieties identified from the 3,493 uniquely identified wheat grain samples. Farmer reported names matched for 28% of the total samples and 21 variety types. This shows that there are clear discrepancies between farmer-reported names and official names of the varieties the underlying cause of which is worth investigating. Moreover, 15% of the farmers reported these samples as ‘improved’ variety for which they could not attach a specific registered name. Interestingly, about 28% of the farmers wrongly reported varieties as ‘local’. Of the variety names matched between farmers’ report and DNA FP identified, best matching varieties were *Mada-Walabu* (70.4%), *Ogolcho* (69.8%), *Tusie* (68.6%), *Digalu* (59.1%) and *Hidasie* (55.3%). Most popularly grown wheat variety from the total sample was *Kakaba* (28.9%). However, from 1019 samples identified as *Kakaba* by DNA FP, only 361 (35.4%) of them were correctly identified by farmers who grew *Kakaba*. Almost all varieties were variously reported by some farmers as *‘local’*. Varieties that were most commonly reported as *‘local’* include: *Enkoy* (66.7%), *Galema* (65.6%), *K6295-4A* (63.6%), and *Arendato* (60.4%). Both *Enkoy* and *Arendato* are old varieties released in 1970s. As variety name of K6295-4A is breeder’s code, it might be difficult for a farmer to remember by heart such unfamiliar and complicated code. Results in Table 3 show that, from the most popularly grown varieties, there is no single variety that was 100% correctly identified by the farmers growing that variety. Wheat variety identification by farmers therefore remains an undeniable challenge in the Ethiopian wheat system.

**Table 3:** Relation between DNA FP identified and farmer reported wheat variety names, Ethiopia 2016/17.

DNA FP identified *(*^*d*^ *= Durum)*		Farmers reported										Not reported by specific name, and neither as ‘improved’ nor ‘local’	
				Reported with specific name				Reported with no specific name but generically as					
		Match		in registry		not in registry		‘improved but name unknown’		‘local but name unknown’			
Name	Freq.	Freq.	*%*	Freq.	*%*	Freq.	*%*	Freq.	*%*	Freq.	*%*	Freq.	*%*
KAKABA	1,019	361	*35*.*4*	182	*17*.*9*	28	*2*.*7*	215	*21*.*1*	224	*22*.*0*	9	*0*.*9*
KUBSA	488	88	*18*.*0*	133	*27*.*3*	26	*5*.*3*	99	*20*.*3*	141	*28*.*9*	1	*0*.*2*
DANDA'A	375	119	*31*.*7*	81	*21*.*6*	21	*5*.*6*	63	*16*.*8*	85	*22*.*7*	6	*1*.*6*
DIGALU	302	172	*57*.*0*	51	*16*.*9*	8	*2*.*6*	10	*3*.*3*	50	*16*.*6*	11	*3*.*6*
BOBICHO	218			51	*23*.*4*	28	*12*.*8*	39	*17*.*9*	99	*45*.*4*	1	*0*.*5*
GALEMA	187	3	*1*.*6*	34	*18*.*2*	9	*4*.*8*	16	*8*.*6*	118	*63*.*1*	7	*3*.*7*
HIDASIE	114	63	*55*.*3*	27	*23*.*7*	1	*0*.*9*	10	*8*.*8*	13	*11*.*4*		
OGOLCHO	97	67	*69*.*1*	24	*24*.*7*					5	*5*.*2*	1	*1*.*0*
PAVON-76	92	30	*32*.*6*	39	*42*.*4*	1	*1*.*1*	5	*5*.*4*	17	*18*.*5*		
ARENDATO ^d^	94			10	*10*.*6*	17	*18*.*1*	9	*9*.*6*	55	*58*.*5*	3	*3*.*2*
SIMBA	69	3	*4*.*3*	28	*40*.*6*			5	*7*.*2*	32	*46*.*4*	1	*1*.*4*
HAWI	62	6	*9*.*7*	19	*30*.*6*	1	*1*.*6*	14	*22*.*6*	21	*33*.*9*	1	*1*.*6*
SHORIMA	45	14	*31*.*1*	15	*33*.*3*			6	*13*.*3*	9	*20*.*0*	1	*2*.*2*
K6295-4A	41	2	*4*.*9*	12	*29*.*3*	2	*4*.*9*	2	*4*.*9*	20	*48*.*8*	3	*7*.*3*
TUSIE	35	24	*68*.*6*	9	*25*.*7*			2	*5*.*7*				
HULLUKA	33	9	*27*.*3*	14	*42*.*4*	1	*3*.*0*	1	*3*.*0*	7	*21*.*2*	1	*3*.*0*
DERESELIGN	30			5	*16*.*7*	6	*20*.*0*	6	*20*.*0*	13	*43*.*3*		
SIRBO	30			18	*60*.*0*	3	*10*.*0*	3	*10*.*0*	5	*16*.*7*	1	*3*.*3*
MADA- WALABU	27	19	*70*.*4*	3	*11*.*1*	1	*3*.*7*			4	*14*.*8*		
ET-13A2	24	1	*4*.*2*	6	*25*.*0*	4	*16*.*7*	3	*12*.*5*	10	*41*.*7*		
BOLLO	23			14	*60*.*9*	1	*4*.*3*	1	*4*.*3*	6	*26*.*1*	1	*4*.*3*
GA’AMBO	22			2	*9*.*1*	11	*50*.*0*	1	*4*.*5*	8	*36*.*4*		
ENKOY	18	2	*11*.*1*	3	*16*.*7*			1	*5*.*6*	12	*66*.*7*		
SENKEGNA	15			1	*6*.*7*	3	*20*.*0*	3	*20*.*0*	8	*53*.*3*		
K6290 BULK	13			4	*30*.*8*	6	*46*.*2*			3	*23*.*1*		
KATAR	14			3	*21*.*4*	3	*21*.*4*			7	*50*.*0*	1	*7*.*1*
ABOLA	11			3	*27*.*3*	2	*18*.*2*			5	*45*.*5*	1	*9*.*1*
SOFUMAR	8	3	*37*.*5*	4	*50*.*0*			1	*12*.*5*				
DASHEN	7			1	*14*.*3*	4	*57*.*1*	2	*28*.*6*				
LASTA ^d^	6			3	*50*.*0*	1	*16*.*7*			2	*33*.*3*		
DAMBAL	4			4	*100*								
MITIKE	3			3	*100*								
LEMU	2			2	*100*								
MANGUDO ^d^	2	1	*50*.*0*					1	*50*.*0*				
TATE ^d^	2	1	*50*.*0*	1	*50*.*0*								
YERER ^d^	2			1	*50*.*0*					1	*50*.*0*		
BIQA	1									1	*100*		
EJERSA ^d^	1	1	*100*										
GUNA	1					1	*100*						
KINGBIRD	1					1	*100*						
KULKULU	1									1	*100*		
LAKECH	1					1	*100*						
MEKELLE-02	1			1	*100*								
TAY	1			1	*100*								
WERER-2 ^d^	1							1	*100*				
Not classified	228			55	*24*.*1*	56	*24*.*6*	38	*16*.*7*	74	*32*.*5*	5	*2*.*2*
Total	3,771	989	*26*.*2*	867	*23*.*0*	247	*6*.*5*	557	*14*.*8*	1,056	*28*.*0*	55	*1*.*5*

Based on varieties identified by DNA FP, 47.3% was covered by varieties released since 2006 (based on a sample of 1,115.7 ha of wheat). Those released before 1996, i.e., above 20 years of age since released, covered 27.3% of the sample area. Results from Eq (2) imply that farmers were growing old varieties., The area weighted average age of wheat varieties identified by DNA FP during the 2016/17 main season was 14 years. This is close to the average 12.6 years estimate made by [27] based on data from eight sub-Saharan African countries. Limited access to new varieties could be the main driver for the farmers to keep old varieties and grow them for long time. As indicated in Table 4, about 50% of the sample wheat area was covered by three popular varieties (*Kakaba*, *Kubsa* and *Danda’a*). During the 2010/11 main production season, there was a serious yellow rust epidemic that devastated wheat production in the most wheat growing regions of the country. Though the government has made lots of efforts in seed production and dissemination of resistant varieties to replace these susceptible varieties by the resistant ones (*Kakaba*, *Danda’a* and *Digalu*), the wider existence of *Kubsa* variety in farmers’ field after such incidence could show that farmers still had some sort of preference for *Kubsa* due to some good reason(s) including higher and stable yield under normal season.

**Table 4:** Popular wheat varieties grown and area share (as identified by DNA FP, 2016/17, aggregate wheat sample area 1,115.7 ha).

Variety	Year released	Share in wheat area %	Cumulative share %
Kakaba	2010	27.0	27.0
Kubsa	1994	13.4	40.4
Danda'a	2010	10.0	50.4
Digalu	2005	9.0	59.3
Bobicho	2002	4.6	64.0
Galema	1995	4.5	68.5
Pavon-76	1982	3.4	71.9
Ogolcho	2012	3.4	75.3
Arendato	1967	3.2	78.4
Hidassie	2012	2.7	81.2
Simba	1999	2.1	83.2
Tusie	1997	2.0	85.3
Hawi	1999	1.9	87.1
Huluka	2012	1.6	88.7
Sirbo	2001	1.5	90.2
Mada Walabu	1999	1.4	91.5
Bolo	2009	1.0	92.5
Others (22 bread and 6 durum varieties)	1967–2016	7.5	100.0

### Explaining varietal identification

As indicated above, there was only 28% match between farmer-reported and official names. Considering observations with matched varietal identification as 1 and those not matched as 0, a binary Logit model was estimated to assess household, farm and seed source characteristics explaining the likelihood that a farmer uses the same name for the wheat variety (s)he grows as in the Official registration. Estimation results in Table 5 show that model farmers and those with better education are more likely in reporting varieties they grow accurately. Moreover, it is less likely that farmers report variety names with their original names with an increase in the years a variety has been recycled and used. In addition, very old wheat varieties are relatively less likely to be accurately identified by farmers. Farmers obtained the initial seeds of the wheat varieties they reported from diverse sources: from cooperatives (23%), seed company (5%), known farmers (26%), from market (17%) and other sources (30%). Compared to seeds initially purchased from cooperatives, wheat seeds obtained from unidentified sources are less likely to be correctly identified by farmers. Labelling seeds in formal seed markets could help farmers to know varietal names for identification. Varieties grown on larger plots are more likely to be identified by farmers. Farmers who grow wheat on larger plots tend to be commercial [21] and are likely to give due attention to the identity of varieties they grow. There is a regional variation in the level of varietal identification. Compared to farmers in Oromia Region, farmers in Tigray, Amhara, and SNNP Regions are lesser chance of identifying varietal names as stated in the national registry. This could be associated with most popular wheat varieties identified in this study having Oromo Language names when released and might take locally adapted names when disseminated to other regions.

**Table 5:** Factors explaining match between DNA FP identified and farmer reported variety names, Ethiopia 2016/17 (Logit estimation).

Explanatory variables	Coefficient.	CSE.	Marginal effect
Male respondent farmer *(dummy*, *1 = yes)*	-0.078	0.157	-0.011
Age of the respondent farmer *(years)*	0.001	0.004	0.000
Education of the respondent farmer *(years of schooling)*	0.081***	0.022	0.012
Model farmer (*dummy*: *1 = yes*)	0.265**	0.128	0.038
Age of the variety in farmer’s hand (*years*)	-0.148***	0.029	-0.021
Number of years since the specific variety was released	-0.084***	0.007	-0.012
Seed obtained from seed company ^a^ (*dummy*: *1 = yes*)	-0.016	0.363	-0.002
Seed obtained from known farmer ^a^ (*dummy*: *1 = yes*)	-0.130	0.171	-0.020
Seed obtained from market (traders/unknown farmers) ^a^ (*dummy*: *1 = yes*)	-0.470**	0.198	-0.069
Seed obtained from other sources ^a^ (*dummy*: *1 = yes*)	-0.390**	0.168	-0.058
Wheat area the specific variety was grown to (*ha*)	0.480***	0.149	0.069
Number of wheat plots per household during the specific season	-0.077	0.064	-0.011
Tigray Region ^*b*^ *(dummy*: *1 = yes)*	-3.041***	0.300	-0.440
Amhara Region ^*b*^ *(dummy*: *1 = yes)*	-1.714***	0.147	-0.248
SNNP Region ^*b*^ *(dummy*: *1 = yes)*	-1.524***	0.156	-0.221
Constant	1.514***	0.321	
*No*. *of Observations*	*2*,*321*		
*LR Chi*^*2*^*(12)*	*625*.*3*		
*Prob > chi*^*2*^	*0*.*000*		
*Pseudo R*^*2*^	*0*.*233*		
*Log likelihood*	*-1028*.*32*		

CSE is Clustered Standard Error at Enumeration Area level

***and ** are significant at 1% and 5% level, respectively.

^a^Seed from Cooperatives is a reference.

^b^Oromia Region is a reference.

Varieties not accurately and uniquely identified by farmers were given different types of names. Some farmers reported using other existing improved variety names, others reported generically as ‘*improved*’ or ‘*local*’, others with a specific but unregistered name. Using these four mismatch categories, and clustering the standard errors at Enumeration Area level, we estimated a multinomial logit regression to identify key factors explaining the likelihood that farmers tended to report using these mismatching names (Table 6). Results show that, in most of the cases, respondents with better education are less likely in picking non-matching variety names. Increase in the duration a variety was recycled and used by a farmer was positively associated with the likelihood that a farmer misreports the variety name. The likelihood of reporting these varieties with non-registered locally adapted names is even higher. A similar situation is observed for varieties released relatively long time ago. Seed sources show mixed results in explaining why a given variety was reported by farmers with a different name not matching to the name in the registry. Compared to seeds purchased from cooperatives, seeds obtained from markets (i.e., from seed traders and unknown farmers in market) are more likely to be reported as ‘*local*’ or with a locally adapted name. Relatively, the likelihood that a given variety is reported as ‘*improved’* or ‘*local*’ is lower when a variety is grown on larger wheat plots. Larger wheat areas are likely to result in more wheat surplus and wheat marketed at the household level, and farmers are more likely to give due attention to variety names. As indicated earlier, compared to farmers from Oromia Region, on average, farmers from the other three Regions (Tigray, Amhara, and SNNP) were more likely to misidentify varieties and report them with names not in the registry, or with generic names as ‘*improved’* or ‘*local’*.

**Table 6:** Factors explaining divergence between DNA FP identified and farmer reported variety names, Ethiopia 2016/17 (Multinomial logit with Clustered Standard errors at Enumeration Area level).

Explanatory variables	Reported with specific but different name				Reported generically as			
	in registry		not in registry		‘*improved’*		*‘local’*	
	Coeff.	CSE	Coeff.	CSE	Coeff.	CSE	Coeff.	CSE
Male respondent farmer *(dummy*: *1 = yes*,*)*	0.075	0.190	0.063	0.300	-0.061	0.283	0.149	0.229
Age of the respondent farmer *(years)*	0.000	0.005	-0.009	0.008	0.001	0.007	0.000	0.006
Education of the respondent farmer *(years of schooling)*	-0.090***	0.030	-0.137**	0.056	-0.043	0.046	-0.075*	0.036
Model farmer *(dummy*: *1 = yes)*	-0.113	0.185	-0.227	0.326	-0.291	0.288	-0.446*	0.240
Age of the variety in farmer’s hand *(years)*	0.091**	0.045	0.219***	0.043	0.116*	0.051	0.215***	0.042
Number of years since the specific variety was released	0.074***	0.012	0.105***	0.014	0.078***	0.013	0.097***	0.013
Seed obtained from seed company ^a^ (*dummy*: *1 = yes*)	-0.041	0.474	1.333**	0.612	-0.528	0.785	0.001	0.622
Seed obtained from known farmer ^a^ (*dummy*: *1 = yes*)	-0.169	0.249	0.752*	0.394	-0.464	0.322	1.144***	0.341
Seed obtained from market (traders/unknown farmers) ^a^ (*dummy*: *1 = yes*)	0.057	0.292	1.304***	0.459	-0.708*	0.398	1.729***	0.400
Seed obtained from other sources ^a^ (*dummy*: *1 = yes*)	0.094	0.266	0.765*	0.442	-0.507	0.365	1.558***	0.369
Wheat area the specific variety was grown to *(ha)*	-0.177	0.199	-0.975**	0.408	-1.641***	0.486	-1.142***	0.382
Number of wheat plots per household during the specific season	0.105	0.089	0.159	0.122	0.207	0.136	0.032	0.111
Tigray Region ^*b*^ *(dummy*: *1 = yes)*	2.007***	0.455	2.952***	0.633	4.668***	0.566	3.315***	0.519
Amhara Region ^*b*^ *(dummy*: *1 = yes)*	0.637	0.314	1.762***	0.500	3.020***	0.458	2.577***	0.378
SNNP Region ^*b*^ *(dummy*: *1 = yes)*	0.688**	0.266	0.890	0.552	2.116***	0.515	2.466***	0.370
Constant	-1.640***	0.459	-4.498***	0.664	-3.408***	0.608	-4.183***	0.605
*Observations*	*2320*							
*Wald Chi2(60)*	*447*.*55*							
*Prob > chi*^*2*^	*0*.*000*							
*Pseudo R*^*2*^	*0*.*1873*							
*Log likelihood*	*-2801*.*54*							

***, ** and * are significant at 1%, 5%, and 10% level, respectively.

^a^ With reference to seeds purchased from Cooperatives.

^b^ Reference to samples from Oromia Region.

### Bias in reporting

When variety names are not actually known by farmers, or varieties were sold to farmers with different names compared to the one in the registry, there could be some possibilities that farmers report old varieties with the names of recently released ones. This could be more likely in marketing when recently released varieties might have better traits that old varieties might not have. The contrary case would also be possible when farmers value traits of an old variety and new varieties could be marketed with the name of the old one. As recently released wheat varieties are usually better than the old ones in many features including resistance to disease and yield performance [28], farmers’ misidentification is ‘*False-Negative*’ when recently released varieties are misidentified and reported with names of old varieties, e.g. a farmer reporting a given variety grown as *Kakaba* (released in 2010) when DNA FP identified it as *Kubsa* (released in 1994), and ‘*False-Positive*’ when actually old varieties are misidentified and reported with names of recently released varieties.

As misidentification increases with age of varieties since released (Table 5), we applied inverse probability weights (IPW) to varietal age and run a multinomial logit model in identifying factors explaining these two biases in misidentified wheat variety names (Table 7). Accordingly, the likelihood of reporting ‘*False-Positive’* increases with age of respondents and number of years since the specific variety was released. The likelihood that farmers confuse names of old varieties with names of relatively younger varieties increases with the number of years since the varieties were released. Compared to seeds obtained from Cooperatives, farmers are more likely to report old varieties obtained from seed companies using names of recently released varieties and vice versa. Accounting for all factors included in this estimation, farmers who grew wheat on relatively larger plots were less likely to report recently released varieties they grew using names of relatively older varieties. The implication for ‘*False-Negative’* is that farmers tend to replace the recently released varieties at hand with something else thinking that what they have at hand is not a recent release. Contrarily, ‘*False-Positive’* implies that farmers might be hesitant to change the actually old variety at hand thinking that it is a recently released variety. Such kind of misinformed knowledge is more serious when a farmer grows rust susceptible varieties thinking that his/her varieties are resistant ones.

**Table 7:** Multinomial logit estimation explaining the bias in reporting improved wheat varieties grown, Ethiopia 2016/17.

Explanatory variables	Though old, farmer wrongly reported it with a name of recently released variety		Though recent, farmer wrongly reported it with a name of variety released long ago	
	*(False-positive)*		*(False-negative)*	
	*N = 309*		*N = 436*	
	Coeff.	CSE	Coeff.	CSE
Male household head *(dummy*: *1 = yes)*	-0.238	0.238	0.109	0.215
Age of the household head *(years)*	0.012*	0.006	0.002	0.006
Education of the household head *(years of schooling)*	-0.019	0.039	-0.057	0.035
Household head is a model farmer *(1 = yes)*	-0.416	0.265	-0.247	0.227
Number of years since the specific variety was released	0.140***	0.017	0.050***	0.017
*Seed purchased (obtained) from*:				
Seed company ^a^ *(dummy*: *1 = yes)*	0.763*	0.461	-1.117*	0.600
Known farmers ^a^ *(dummy*: *1 = yes)*	-0.059	0.306	0.084	0.287
Market (traders/unknown farmers) ^a^ *(dummy*: *1 = yes)*	0.389	0.305	0.068	0.316
Seed from other sources ^a^ *(dummy*: *1 = yes)*	-0.226	0.321	0.106	0.306
Plot area the specific variety was grown to *(ha)*	0.197	0.337	-1.083**	0.440
Number of wheat plots per household during the specific season	0.133	0.143	0.077	0.136
Tigray Region ^b^ *(dummy*: *1 = yes)*	1.204**	0.515	1.458***	0.543
Amhara Region ^b^ *(dummy*: *1 = yes)*	0.417	0.404	0.908**	0.392
SNNP Region ^b^ *(dummy*: *1 = yes)*	1.739***	0.334	0.512	0.376
Constant	-4.451***	0.553	-1.680***	0.591
*Observations*	*1*,*290*			
*Wald Chi*^*2*^*(24)*	*140*.*76*			
*Prob > Chi*^*2*^	*0*.*000*			
*Pseudo R2*	*0*.*151*			
*Log likelihood*	*-1064*.*79*			

***, ** and * are significant at 1%, 5%, and 10% level, respectively.; Exact match is considered as a reference category; CSE is Clustered Standard Error at Enumeration Area level;

^a^ with reference to seeds purchased from Cooperatives.

^b^ Reference to Oromia Region.

### Estimating improved wheat variety adoption

Using the conventional household survey method (and without considering the number of seasons wheat seed was recycled), our data suggests an improved wheat varietal adoption rate of 50–67% by the number of wheat plots and 61–73% by wheat area. The lower bound of the ranges consider only registered varietal names and the upper bound includes generic names. Interestingly, 93% of the varieties that farmers reported as “name unknown but local” were identified by DNA FP as improved varieties (Table 3). DNA FP takes the adoption estimate to 95% in terms of wheat area (Table 8). Still, some caveats are in order, not least as some of the identified improved wheat varieties were released 30–40 years ago, and it is about 20–30 years since their formal seed multiplication was stopped.

**Table 8:** Improved wheat variety adoption estimates using farmers’ report and DNA FP data, Ethiopia 2016/17.

Category	Number of plots				Wheat area			
	Farmer reported		DNA FP identified		Farmer reported		DNA FP identified	
	Freq.	*%*	Freq.	*%*	ha	%	ha	%
Identified with registered specific name	1957	*50*.*4*	3543	*94*.*0*	452	*60*.*8*	692.1	*94*.*9*
Reported as *‘improved’*& not in registry	634	*16*.*3*		* *	88	*11*.*8*		* *
Reported as *‘local’* & not in registry	1257	*32*.*4*		* *	196	*26*.*4*		* *
Not identified	36	*0*.*9*	228	*6*.*0*	7	*1*.*0*	37.0	*5*.*1*
Total	3884		3771		743.3		729.1	* *

Our analysis thereby highlights that neither the farmers’ or the DNA FP reports provide a complete picture of the adoption status of improved wheat varieties. Instead, they might need to complement each other. While the household surveys can shed light on the history of the varieties grown, the duration of varietal use and contextual factors, the DNA FP (only) accurately identifies the variety. The misidentification by farmers is unlikely intentional–and may reflect that farmers have incomplete or incorrect information of the varieties they are growing including the actual names assigned to the specific varieties by breeders when released. Although it is advisable to use these two approaches as a potential complement in varietal identification as well as adoption and impacts assessment, the costs involved in data collection and DNA sequencing should not be underestimated. Advanced methods including hand-held portable devices (technologies) could help in identifying crop varieties in the field or from grain samples with acceptable levels of accuracy and reduce costs [29]. In general, it is always worth to consider what level of accuracy is expected (or what level of error is allowed) in estimating some of the outcome variables. Here, judgement of the researcher is essential in choosing the data collection method(s) for variety identification and the level of accuracy each method could provide at the data analysis stage.

### Linking varieties to their germplasm sources

Only 50.4% of the farmer-reported varieties had specific names appearing in the variety registry–the other half included generic names or names that did not register (Table 8). On the other hand, DNA FP correctly identified 94% of the samples collected failing to identify only 6%. Focusing on those varieties identified by a specific name both by farmers and DNA FP, the distribution of these sub-samples in terms of their germplasm source is more or less the same. For instance, from the varieties identified by farmers with their specific registered names, 92% were derived from the International Maize and Wheat Improvement Center (CIMMYT) germplasm–although it should be remembered that only half of the farmers were able to report such names (Table 9). Similarly, from those samples identified by DNA fingerprinting, 91% were linked to CIMMYT germplasm. Accounting for the 6% that were not classified by the DNA sequencing, CIMMYT’s share in the national wheat varietal use was 86%. These results show that over 94.5% of all varieties identified by DNA FP were derived from the CGIAR, specifically CIMMYT and ICARDA (International Center for Agricultural Research in the Dry Areas), while those which directly came from the national research system accounted for 3.4%.

**Table 9:** Varietal sources using farmers report and DNA FP identified (by number of wheat plots, n; and by sample wheat area, ha), Ethiopia 2016/17.

Germplasm source	Farmer reported with registered specific name				DNA FP identified			
	n	%	ha	%	n	%	ha	%
CIMMYT	1,805	92.2	417.8	92.5	3,236	91.3	635.1	91.8
Colombia	1	0.1						
Ecuador	9	0.5	1.8	0.4				
Ethiopia	16	0.8	2.4	0.5	122	3.4	23.1	3.3
ICARDA	83	4.2	22.9	5.1	112	3.2	22.3	3.2
Kenya	39	2	6	1.3	72	2	11.1	1.6
USA	1	0.1						
Source unknown	3	0.2	0.7	0.1	1	0	0.4	0.1
Total	1,957	100	452	100	3,543	100	692	100

## Discussions

Understanding crop varietal use is useful to inform policy decisions and agricultural development. For instance, crop varieties differ in their disease and pest tolerance and other traits. Some policies intend to replace old varieties with new and better performing ones; some market development calls for specific crop variety with special traits, etc. In responding to these and similar demands, accurate varietal identification at the plot levels and aggregating varietal distributions at a regional/national or agroecology levels are crucial. The commonly used method in varietal level data collection is farm household survey where farmers report the type of specific varieties they grew during a specific season. However, varietal names collected using this approach have their own challenges including the fact that most farmers report varieties in locally adapted names which is usually different from the original names these varieties had when registered and/or released. Even for those varieties reported with known names in the variety registry book, there is no guarantee that these varieties indeed are what they are reported to be. Farmers may also not necessarily know or give names to every crop varieties they grow. Under such circumstances, using DNA FP with a robust reference library built based on breeders’ seed collected from the institution(s) under which the individual varieties were registered is indispensable.

This paper shows the disparity between estimates of the level of improved wheat variety adoption generated using farmers’ reporting and DNA FP approaches. DNA FP identified 94% of the samples as improved whereas the corresponding household survey estimate was 50–67% (depending on the way we define what farmers reported as improved or not). There are issues in both approaches considered for varietal identification in this study. First, unless supported by further inquiry through surveys, it is difficult to know how long the varieties identified by DNA FP have been actually used in the production system. This is important particularly in situations where improved seeds could lose their yield potential due to mixture in the field when recycled for longer years and need to be replaced with fresh seed for better performance. Second, as shown in this study, of the 1,957 wheat samples reported by farmers with names in the variety registry book (i.e., 50% of the total sample), only half of them (i.e., 989 samples) matched with the variety names identified by DNA FP. This implies that there is sizeable mismatch between varietal names used by farmers and that in the national registry. Such mixing up of improved wheat variety names by farmers makes variety specific results obtained from farmers’ elicitation dubious.

The self-pollinated nature of wheat allows a relatively better purity over a certain period of time compared to cross-pollinated crops. Theoretically, one might thus expect farmers to know and report the actual name of improved wheat varieties. Instead, farmers’ misidentification of the wheat varieties they grow was relatively large (i.e., 66–72%). Several factors associated with the misidentification were identified in this study, including education level of farmers, number of years of seed recycling, whether a farmer is relatively specialized in wheat production and whether the seed was purchased from formal seed sources, etc. Results imply that any study targeted to varietal identification using household survey data based on farmers’ elicitation need to double-check how accurate these varietal names are and put some further inquiries to enhance the level of accuracy. If not, decisions taken based on such kind of studies that relied only on farmers’ elicitation could lead to undesirable outcomes. In this regard, further studies might be relevant to sort out the sources of deviations between names that farmers use in identifying a variety and what actually these varieties are by considering different nodes of the seed development and distribution channels. In addition, to complement surveys conducted to document variety specific data, it is essential to look for advanced methods and technologies that could help in identifying crop varieties in the field or based on grain samples with acceptable levels of accuracy.

## Conclusions

Using DNA FP and farmers’ elicitation approaches in varietal identification, this paper assessed the level of misidentification by smallholder farmers of the wheat varieties they grew and identifying key variables explaining the misidentification. Data showed clear disparity between varietal identification based on farmers’ elicitation and DNA FP methods with important implications for the estimated level of improved wheat variety adoption. DNA FP identified 94% of wheat samples in Ethiopia in 2016/17 as improved whereas the household survey estimated this at 50–67%. Only 28–34% of varieties were correctly identified, i.e. a misidentification of some 70% whereby farmer-reported variety names did not match those identified by DNA FP. Level of education, source of seed, level of seed recycling, age of the variety since release, and number and size of wheat plots determine the ability of farmers to correctly identify the wheat varieties they grow in relation to variety names in the national registry. Our findings imply that variety specific adoption and impact assessments based solely on farmer-reported variety names are highly dubious. Thus, regardless of the associated costs, we recommend DNA FP as a reliable method in varietal identification. Still, DNA FP remains mute in the identification of contextual and explanatory factors and thus is best enriched with complementary targeted household survey data. In combination, they can provide sound and reliable varietal adoption and impact assessments, and generate useful knowledge to inform policy recommendations related to varietal replacement and seed systems development.
